# Supplementary material for: Bst2-targeted senotherapy restores visual function by eliminating senescent retinal cells
Source: Nat Commun. 2026 Mar 18;17:4135. doi: 10.1038/s41467-026-70797-2 (PMC13149862; doi:10.1038/s41467-026-70797-2)
Supplement: Supplementary file 1 — Supplementary Information [file 41467_2026_70797_MOESM1_ESM.pdf]

Supplementary Information

Bst2-Targeted Senotherapy Restores Visual Function by Eliminating Senescent Retinal Cells

Jun Yong Oh[1,†], Jae-Byoung Chae[2,†], Hyo Kyung Lee[3], Chul-Woo Park[2], Minseo Bae[2], Gyuri Kim[4], Yujeong Oh[4], Gyeongseok Yang[1,5], Sangpil Kim[1], Hae Won Ok[1], Dohyun Kim[1], Chaekyu Kim[6], Semin Lee[3], Jiwon Jang[4], Hyewon Chung[2,7,]* and Ja-Hyoung Ryu[1,5,]*

[1]*Department of Chemistry, Ulsan National Institute of Science and Technology (UNIST), Ulsan 44919, Republic of Korea*

[2]*Department of Ophthalmology, Konkuk University College of Medicine, Seoul, Republic of Korea.*

[3]*Department of Biomedical Engineering, College of Information and Biotechnology, Ulsan National Institute of Science and Technology (UNIST), Ulsan 44919, Republic of Korea.*

[4]*Department of Life Sciences, Pohang University of Science and Technology (POSTECH), Pohang, Korea*

[5]*GIST InnoCORE AI-Nano Convergence Initiative for Early Detection of Neurodegenerative Diseases, Gwangju Institute of Science and Technology, 61005 Gwangju, Republic of Korea*

[6]*Fusion Biotechnology, Inc. Ulsan 44919, Republic of Korea.*

[7]*Department of Ophthalmology, Konkuk University Medical Center, Seoul, Republic of Korea.*

[†] These authors are equally contributed

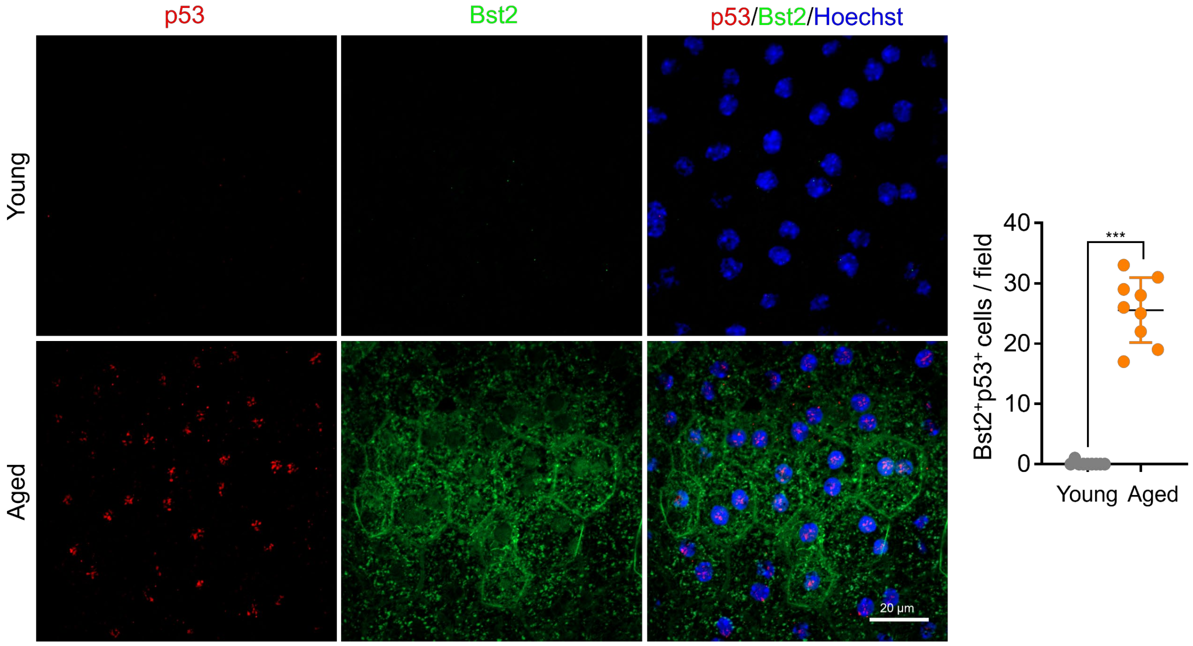

Supplementary Figure 1. Senescence-associated Bst2 upregulation in aged RPE. Representative immunofluorescence images of Bst2 and p53 in RPE flat mounts from young (3-month-old) and naturally aged (24-month-old) mice. Scale bar: 20 µm. Quantification shows the number of $Bst2^{+}p53^{+}$ cells per field. Data are presented as mean ± SD. Statistical significance was determined using a two-tailed unpaired t-test; $***P < 0.001$.

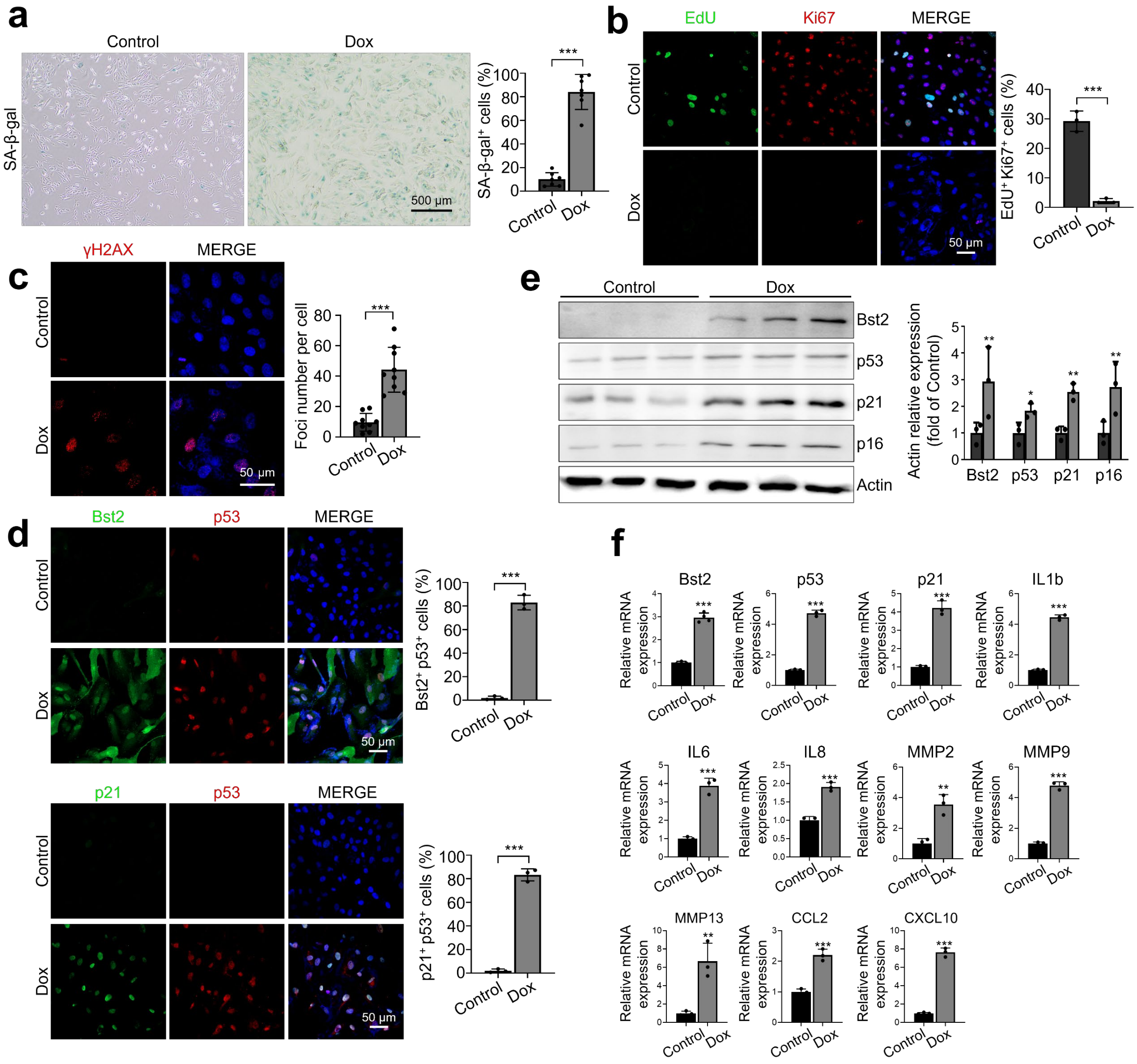

Supplementary Figure 2. Validation of doxorubicin (Dox)-induced senescence in ARPE-19 cells. ARPE-19 cells were treated with doxorubicin (Dox) for 3 days, followed by washout and culture in drug-free medium for an additional 4 days (day 7 endpoint). (a) Representative SA-β-gal staining images and quantification of SA-β-gal–positive cells. (b) EdU incorporation and Ki67 immunostaining showing loss of proliferative activity in Dox-treated cells after washout. (c) Representative γH2AX immunofluorescence images and quantification of γH2AX foci per nucleus. (d) Representative immunofluorescence images of p53 and p21 (left) and Bst2 and p53 (right); corresponding quantification is provided. (e) Immunoblot analysis of senescence markers (p53, p21, p16) and Bst2 with densitometric quantification (normalized to actin). (f) qRT-PCR analysis showing increased expression of Bst2 and selected SASP-associated transcripts in Dox-treated cells. Scale bars: 50 μm (b–d). Two-tailed unpaired t-test were used for statistical analysis of the data shown. The data are presented as the mean ± SD. * $P < 0.05$, ** $P < 0.01$, and *** $P < 0.001$ by a t-test.

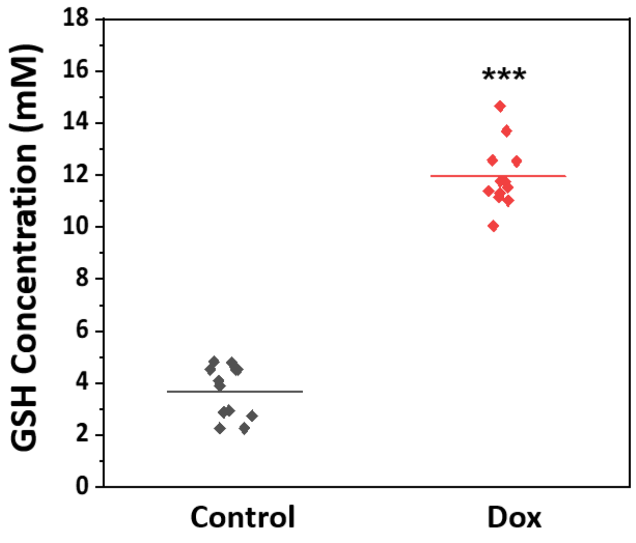

Supplementary Figure 3. Intracellular glutathione (GSH) levels in normal and Dox-induced ARPE-19 cells. Intracellular GSH concentrations were measured using a quantitative GSH assay in normal and Dox-induced senescent ARPE-19 cells. Data are presented as individual data points with mean ± SD. Statistical significance was assessed using an unpaired two-tailed Student's t-test.

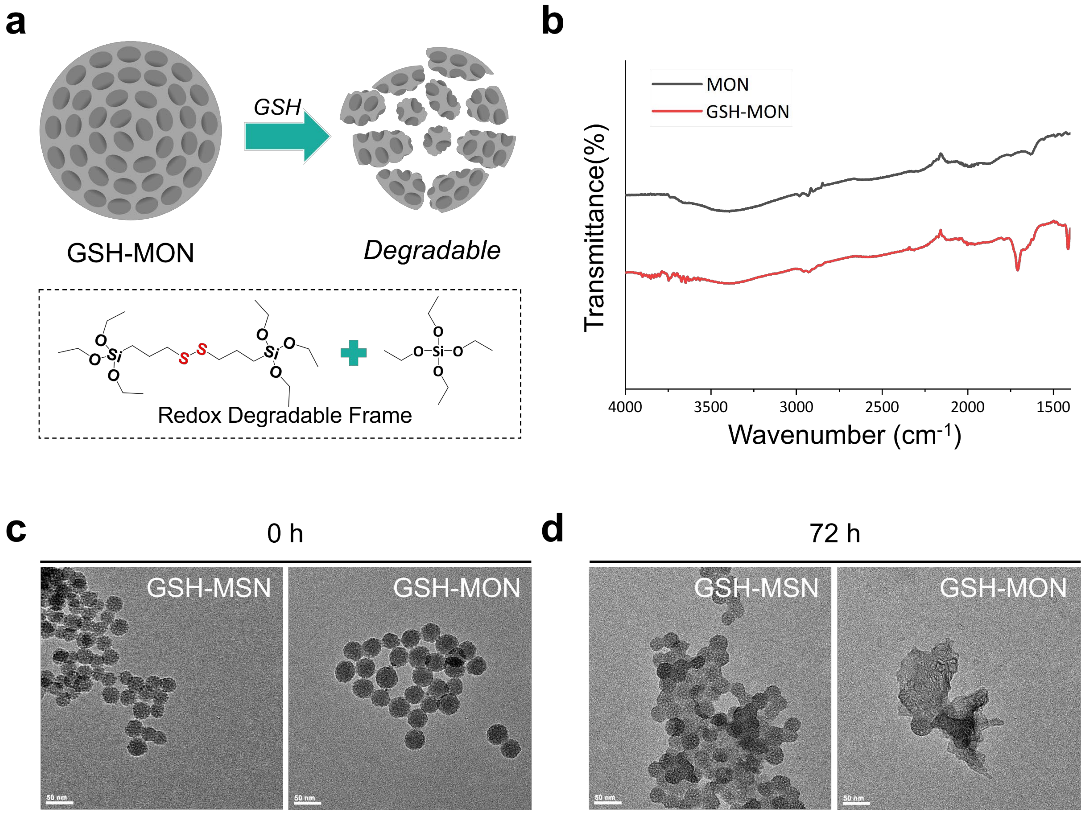

Supplementary Figure 4. GSH-responsive degradability of mesoporous organosilica nanoparticles. (a) Schematic illustration of glutathione-responsive mesoporous organosilica nanoparticles (GSH-MON) incorporating redox-cleavable organosilica backbones, compared with conventional mesoporous silica nanoparticles (GSH-MSN). (b) FT-IR spectra of MON and GSH-MON. Transmission electron microscopy (TEM) images of GSH-MSN and GSH-MON after incubation in neutral PBS containing 10 mM GSH for (c) 0 h and (d) 72 h. Scale bars, 20 nm.

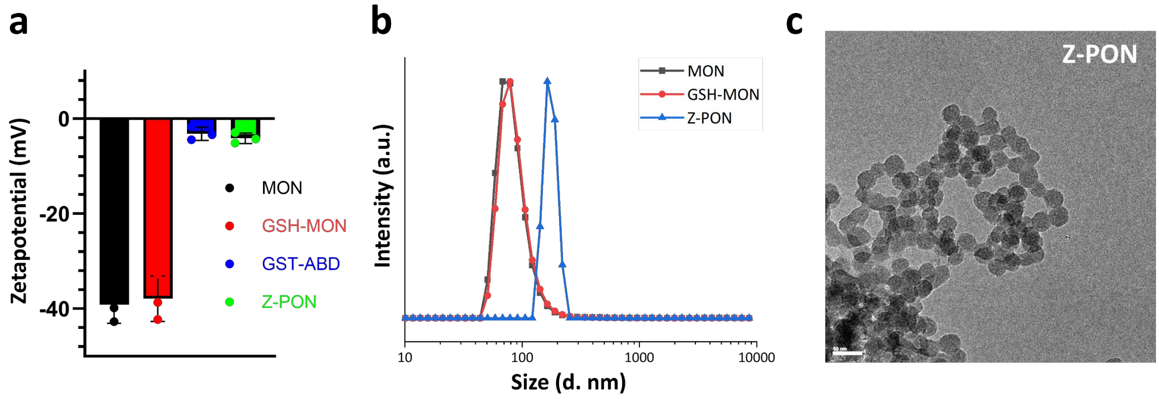

Supplementary Figure 5. Physicochemical characterization of Z-PON particles. (a) Zeta-potential measurements of MON, GSH-MON, GST-ABD, and GST-ABD–modified MON (Z-PON). (b) Hydrodynamic diameter of MON, GSH-MON, and GST-ABD–modified MON (Z-PON) measured by dynamic light scattering. (c) TEM image of Z-PON particles. Scale bar, 20 nm. Data are presented as individual data points with mean ± SD.

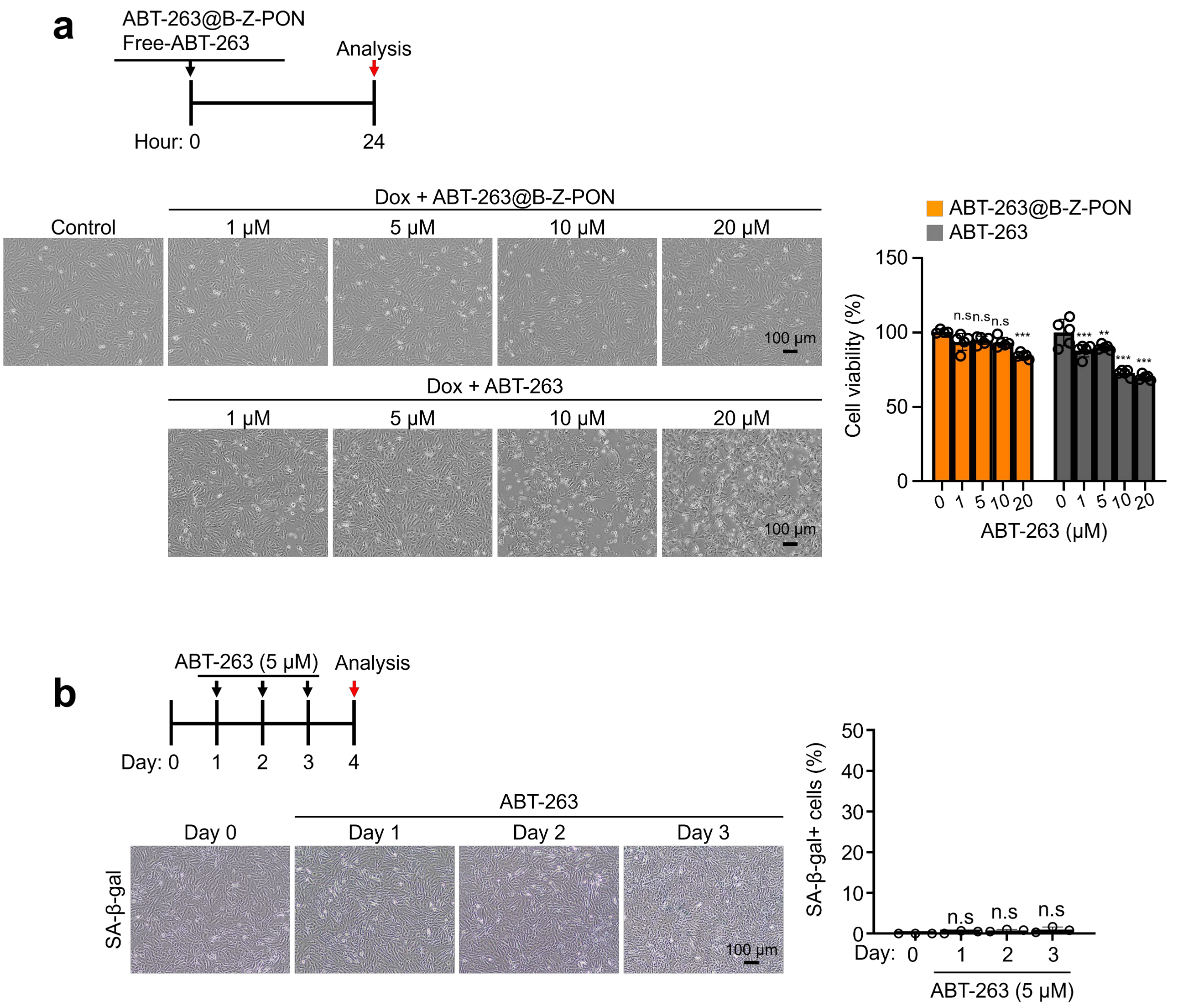

Supplementary Figure 6. Cytotoxicity evaluation of ABT-263@B-Z-PON and free ABT-263 in normal ARPE-19 cells. (a) Representative phase-contrast images of normal ARPE-19 cells treated with ABT-263@B-Z-PON or free ABT-263 at the indicated concentrations (0, 1, 5, 10, and 20 μM). (b) Normal ARPE-19 cells were treated with free ABT-263 (5 μM) once daily for 1, 2, or 3 consecutive days, followed by fixation and SA-β-gal staining. No SA-β-gal–positive cells were detected at any time point under these conditions. Scale bars: 100 μm. Data are presented as mean ± SD. Statistical significance was assessed using one-way ANOVA followed by Tukey's multiple comparisons test. ***P* < 0.01 and ****P* < 0.001.

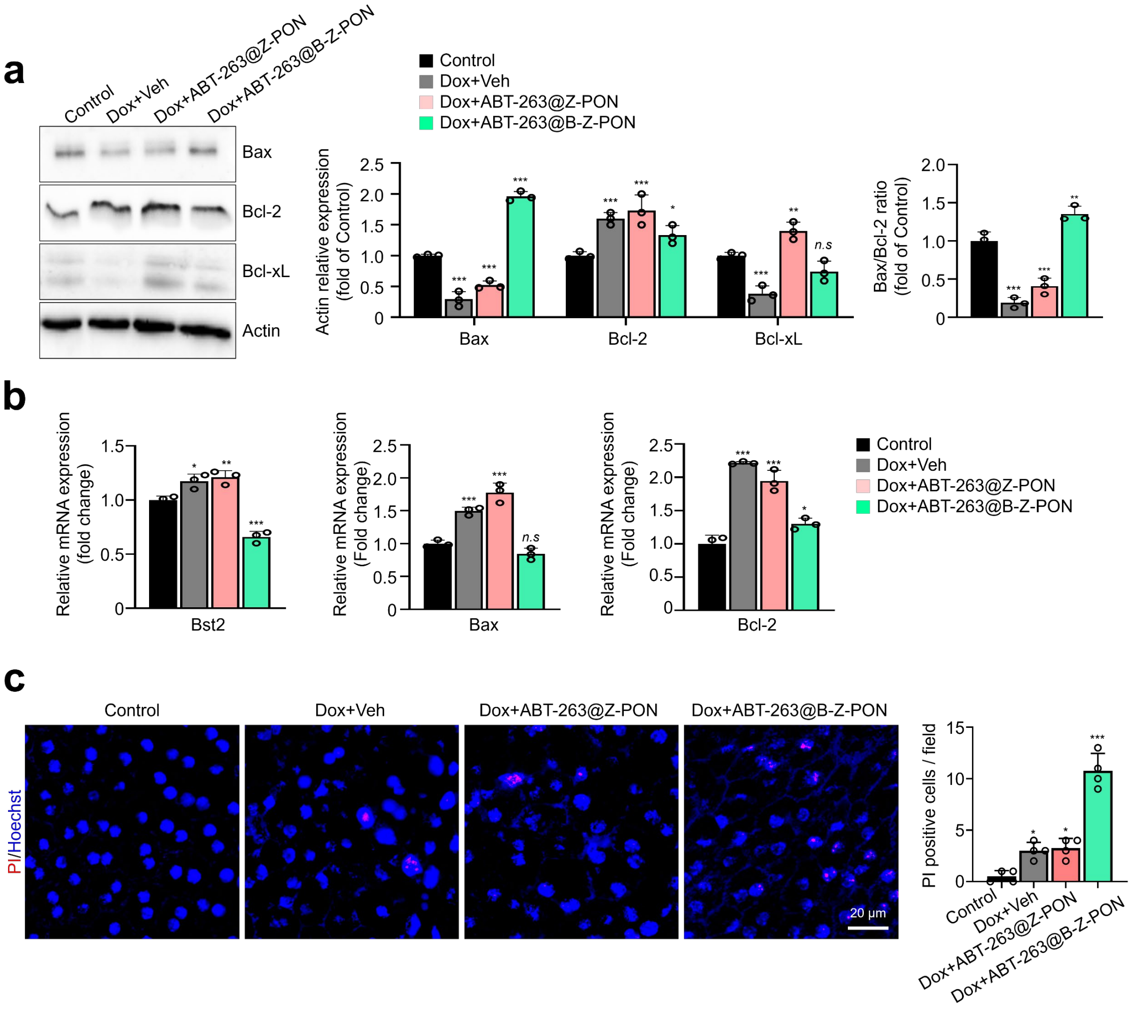

Supplementary Figure 7. Pro-apoptotic signaling and propidium iodide (PI)-positive RPE after Bst2-targeted senolysis in vivo. (a) Immunoblot analysis of Bax, Bcl-2, and Bcl-xL in RPE lysates from vehicle- or Dox-injected eyes treated with vehicle, ABT-263@Z-PON, or ABT-263@B-Z-PON. Densitometric quantification and Bax/Bcl-2 ratios are shown. (b) qRT–PCR analysis of Bst2, Bax, and Bcl-2 mRNA expression under the indicated conditions. (c) Representative propidium iodide (PI) staining of RPE flat mounts, indicating loss of membrane integrity in dying or dead cells, with quantification of PI-positive RPE cells per field. Scale bar: 20 μm. One-way ANOVA followed by Tukey's multiple comparisons test was used. Data are presented as mean ± SD. **P* <0.05, ***P* < 0.01, and ****P* < 0.001 by a t test.

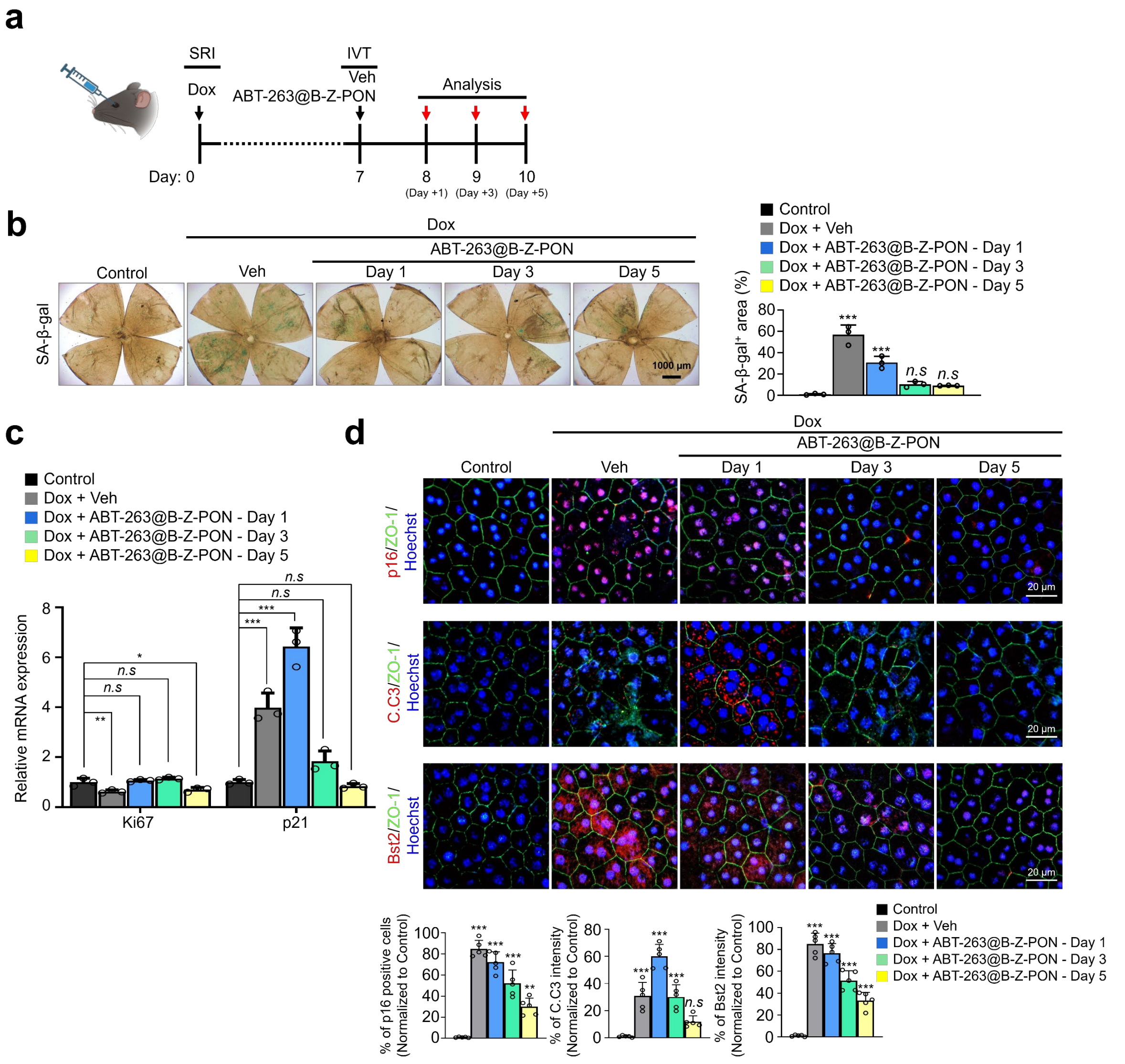

Supplementary Figure 8. Time-dependent Bst2-targeted clearance of Dox-induced senescent RPE cells in vivo. (a) Schematic of the experimental timeline, including subretinal Dox injection to induce RPE senescence, intravitreal ABT-263@B-Z-PON administration 7 days after Dox, and analyses at the indicated time points. (Created in BioRender. Oh, J. Y. (2026) https://BioRender.com/vpzyz04) (b) Representative SA-β-gal staining of RPE flat mounts with quantification of SA-β-gal–positive area. Scale bar, 1000 μm. (c) qRT–PCR analysis of Ki67 and p21 mRNA expression levels under the indicated conditions. (d) Representative immunofluorescence images of p16, cleaved caspase-3 (C.C3), Bst2, and ZO-1 with Hoechst counterstaining, with corresponding quantifications. Scale bar: 20 μm. Data are presented as mean ± SD. Statistical analyses were performed using one-way ANOVA with Tukey's multiple-comparisons test. $*P < 0.05$, $**P < 0.01$, $***P < 0.001$; n.s., not significant.

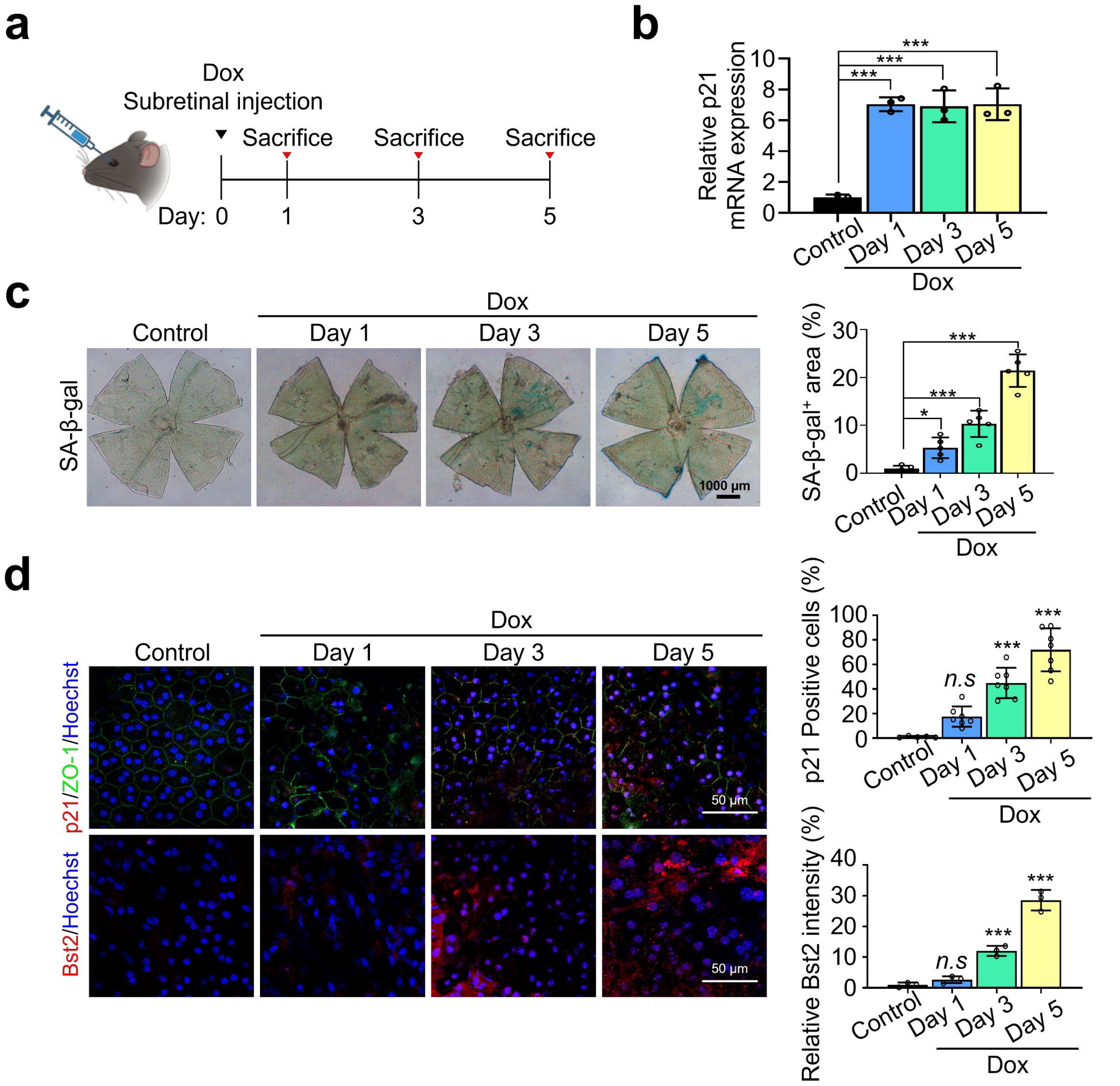

Supplementary Figure 9. Time course of senescence-marker induction after subretinal Dox injection. (a) Schematic of the experimental timeline. (Created in BioRender. Oh, J. Y. (2026) https://BioRender.com/u1j7fju) (b) qRT–PCR analysis of p21 mRNA expression in RPE tissues at the indicated time points. (c) Representative SA-β-gal staining of RPE flat mounts with corresponding quantification of SA-β-gal–positive area. (d) Representative immunofluorescence images of p21 (top) or Bst2 (bottom) with ZO-1 staining and Hoechst counterstaining, and quantification of p21-positive cells. Scale bars: 2000 μm (c), 50 μm (d). One-way ANOVA followed by Tukey's multiple comparisons test was used for statistical analysis of the data shown. The data are presented as the mean ± SD. * $P < 0.05$ and *** $P < 0.001$ from t-test.

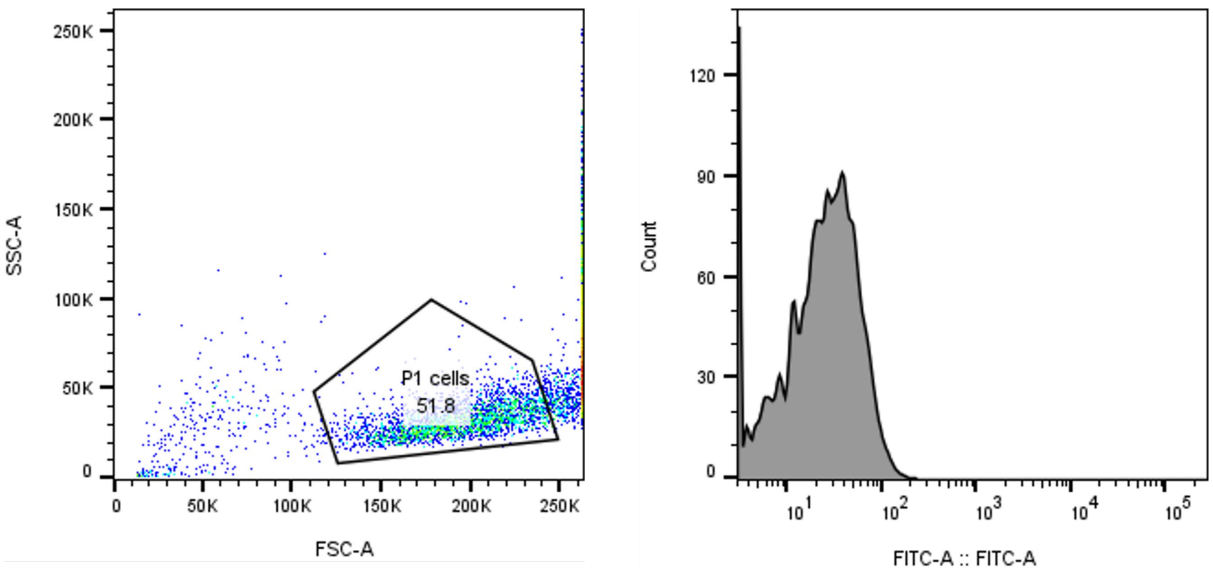

Supplementary Figure 10. Flow cytometry gating strategy. Cells were first gated based on forward scatter area (FSC-A) versus side scatter area (SSC-A) to exclude debris and select the main cell population (P1). Fluorescence intensity (FITC-A) was subsequently quantified from P1-gated cells. All flow cytometry histograms shown in Fig. 2c,d were derived from P1-gated cells.

Supplementary Table 1. List of primers used in the in vivo experiments

Gene	Forward primer (5′–3′)	Reverse primer (5′–3′)
p53	CCAGGATGTTGAGGAGTTTTT	GCTTATTGAGGGGAGGAGAG
p21	ATCCACAGCGATATCCAGAC	CTCCGTGACGAAGTCAAAGT
Bst2	CAAACTCCTGCAACCTGACCGT	CAAACTCCTGCAACCTGACCGT
Bax	AGGATGCGTCCACCAAGAAGCT	TCCGTGTCCACGTCAGCAATCA
Bcl-2	CCTGTGGATGACTGAGTACCTG	AGCCAGGAGAAATCAAACAGAGG
Ki67	GAGGAGAAACGCCAACCAAGAG	TTTGTCCTCGGTGGCGTTATCC
Gapdh	CATCACTGCCACCCAGAAGACTG	ATGCCAGTGAGCTTCCCGTTCAG

Supplementary Table 2. List of primers used in the in vitro experiments

Gene	Forward primer (5′–3′)	Reverse primer (5′–3′)
p53	CCTCAGCATCTTATCCGAGTGG	TGGATGGTGGTACAGTCAGAGC
p21	AGGTGGACCTGGAGACTCTCAG	TCCTCTTGGAGAAGATCAGCCG
IL-1β	CCACAGACCTTCCAGGAGAATG	GTGCAGTTCAGTGATCGTACAGG
IL-6	AGACAGCCACTCACCTCTTCAG	TTCTGCCAGTGCCTCTTTGCTG
IL-8	GAGAGTGATTGAGAGTGGACCAC	CACAACCCTCTGCACCCAGTTT
MMP-2	AGCGAGTGGATGCCGCCTTTAA	CATTCCAGGCATCTGCGATGAG
MMP-9	GCCACTACTGTGCCTTTGAGTC	CCCTCAGAGAATCGCCAGTACT
MMP-13	CCTTGATGCCATTACCAGTCTCC	AAACAGCTCCGCATCAACCTGC
CCL2	AGAATCACCAGCAGCAAGTGTCC	TCCTGAACCCACTTCTGCTTGG
CXCL10	GGTGAGAAGAGATGTCTGAATCC	GTCCATCCTTGGAAGCACTGCA
GAPDH	GTCTCCTCTGACTTCAACAGCG	ACCACCCTGTTGCTGTAGCCAA
