# Supplementary material for: Bst2-targeted senotherapy restores visual function by eliminating senescent retinal cells
Source: Nat Commun. 2026 Mar 18;17:4135. doi: 10.1038/s41467-026-70797-2 (PMC13149862; doi:10.1038/s41467-026-70797-2)
Supplement: Supplementary file 2 — reporting summary [file 41467_2026_70797_MOESM2_ESM.pdf]

nature portfolio

Corresponding author(s): Hyewon Chung, Ja-Hyoung Ryu
Last updated by author(s): Jan 30, 2026

Reporting Summary

Nature Portfolio wishes to improve the reproducibility of the work that we publish. This form provides structure for consistency and transparency in reporting. For further information on Nature Portfolio policies, see our Editorial Policies and the Editorial Policy Checklist.

Statistics

For all statistical analyses, confirm that the following items are present in the figure legend, table legend, main text, or Methods section.

n/a	Confirmed	
☐	☒	The exact sample size (*n*) for each experimental group/condition, given as a discrete number and unit of measurement
☐	☒	A statement on whether measurements were taken from distinct samples or whether the same sample was measured repeatedly
☐	☒	The statistical test(s) used AND whether they are one- or two-sided<br>*Only common tests should be described solely by name; describe more complex techniques in the Methods section.*
☒	☐	A description of all covariates tested
☒	☐	A description of any assumptions or corrections, such as tests of normality and adjustment for multiple comparisons
☐	☒	A full description of the statistical parameters including central tendency (e.g. means) or other basic estimates (e.g. regression coefficient) AND variation (e.g. standard deviation) or associated estimates of uncertainty (e.g. confidence intervals)
☐	☒	For null hypothesis testing, the test statistic (e.g. *F*, *t*, *r*) with confidence intervals, effect sizes, degrees of freedom and *P* value noted<br>*Give P values as exact values whenever suitable.*
☒	☐	For Bayesian analysis, information on the choice of priors and Markov chain Monte Carlo settings
☒	☐	For hierarchical and complex designs, identification of the appropriate level for tests and full reporting of outcomes
☒	☐	Estimates of effect sizes (e.g. Cohen's *d*, Pearson's *r*), indicating how they were calculated

Our web collection on statistics for biologists contains articles on many of the points above.

Software and code

Policy information about availability of computer code

Data collection	Zen Blue confocal software (Zeiss), CFX connect Real-time PCR Detection System (BIO-RAD), Celeris Diagnosys Electroretinography System (CELERIS)
Data analysis	To process, quantify and analyze sequencing data sets, the following tools were used:<br>Cell Ranger v6.0.1<br>R v4.1.1<br>Seurat v4.1.1<br>GraphPad Prism 9.1.0 software<br>Fiji ImageJ<br>Flow Jo 10<br>Origin 2026

For manuscripts utilizing custom algorithms or software that are central to the research but not yet described in published literature, software must be made available to editors and reviewers. We strongly encourage code deposition in a community repository (e.g. GitHub). See the Nature Portfolio guidelines for submitting code & software for further information.

Data

Policy information about availability of data

All manuscripts must include a data availability statement. This statement should provide the following information, where applicable:

- Accession codes, unique identifiers, or web links for publicly available datasets
- A description of any restrictions on data availability
- For clinical datasets or third party data, please ensure that the statement adheres to our policy

The scRNA-seq data used in this study are available in the Gene Expression Omnibus (GEO) database under accession codes GSE282283 (https://www.ncbi.nlm.nih.gov/geo/query/acc.cgi?acc=GSE282283) and GSE183572 (https://www.ncbi.nlm.nih.gov/geo/query/acc.cgi?acc=GSE183572). All other data supporting the findings of this study are available within the article and its Supplementary Information.

Research involving human participants, their data, or biological material

Policy information about studies with human participants or human data. See also policy information about sex, gender (identity/presentation), and sexual orientation and race, ethnicity and racism.

Reporting on sex and gender	N/A
Reporting on race, ethnicity, or other socially relevant groupings	N/A
Population characteristics	N/A
Recruitment	N/A
Ethics oversight	N/A

Note that full information on the approval of the study protocol must also be provided in the manuscript.

Field-specific reporting

Please select the one below that is the best fit for your research. If you are not sure, read the appropriate sections before making your selection.

☒ Life sciences ☐ Behavioural & social sciences ☐ Ecological, evolutionary & environmental sciences

For a reference copy of the document with all sections, see nature.com/documents/nr-reporting-summary-flat.pdf

Life sciences study design

All studies must disclose on these points even when the disclosure is negative.

Sample size	Preliminary experiments were conducted when possible to determine requirements for sample size. Sample size sufficiency was determined by preliminary data or discussion. For statistical significance, the sample size was always independently performed three times or more times.
Data exclusions	No data were excluded from the analysis.
Replication	All experiments were replicated or conducted independently for at least three times.
Randomization	Age-matched animals were randomly allocated to each experimental group. In the studies on the disease models, male mice were considered equivalent and randomly assigned to treatment groups.
Blinding	Blinding Investigators were not blinded when gaining and analyzing data.

Reporting for specific materials, systems and methods

We require information from authors about some types of materials, experimental systems and methods used in many studies. Here, indicate whether each material, system or method listed is relevant to your study. If you are not sure if a list item applies to your research, read the appropriate section before selecting a response.

Materials & experimental systems

n/a	Involved in the study
☐	☒ Antibodies
☐	☒ Eukaryotic cell lines
☒	☐ Palaeontology and archaeology
☐	☒ Animals and other organisms
☒	☐ Clinical data
☒	☐ Dual use research of concern
☒	☐ Plants

Methods

n/a	Involved in the study
☒	☐ ChIP-seq
☐	☒ Flow cytometry
☒	☐ MRI-based neuroimaging

Antibodies

Antibodies used

Used:
Primary antibodies
Bst2 (1:250, Santa Cruz Biotechnology, sc-390719)
p53 (1:500, Santa Cruz Biotechnology, sc-126)
p21 (1:1000, Abcam, ab109520)
p16 (1:1000, Abcam, ab189034)
β-actin (1:5000, Santa Cruz Biotechnology, sc-47778)
Ki67 (1:500, Abcam, ab16667)
Cleaved caspase-3 (1:500, Abcam, ab2302)
ZO-1 (1:1000, Invitrogen, 61-7300, 33-9100)
Bcl-2 (1:500, Abcam, ab32124)
Bax (1:200, Santa Cruz Biotechnology, sc-20067)
Bcl-xL (1:500, Santa Cruz Biotechnology, sc-8392)
p53 (rabbit, 1:500; Cell Signaling Technology, #2527)

Secondary antibodies
Alexa Fluor 488 Goat anti-mouse (1:250, Invitrogen, A32723)
Alexa Fluor 488 Goat anti-Rabbit (1:250, Invitrogen, A32731)
Alexa Fluor 555 Goat anti-mouse (1:250, Invitrogen, A32727)
Alexa Fluor 555 Goat anti-Rabbit (1:250,Invitrogen, A32732)

Validation

All of the antibodies are commercially available, and have been validated by manufacturers for the species and applications used inthis study.
Manufacturers validation statements are described on the following websites:
Bst2 (Santa Cruz Biotechnology, sc-390719) https://www.scbt.com/ko/p/bst-2-antibody-e-4?srsltid=AfmBOoptBJFqiXIQCIDqmcsrN62In2LD9s0OOAskCWZ-MPVcLfMhEyx5
p53 (Santa Cruz Biotechnology, sc-126) https://www.scbt.com/p/p53-antibody-do-1?srsltid=AfmBOoqqNzEEiw4u6ap8jcc_LjI33bOoXjux01EoIikqEYeDusRrDjAA
p21 (Abcam, ab109520) https://www.abcam.com/en-us/products/primary-antibodies/p21-antibody-epr362-ab109520
p16 (Abcam, ab189034) https://www.abcam.com/en-us/products/primary-antibodies/cdkn2a-p16ink4a-antibody-n-terminal-ab189034
β-actin (Santa Cruz Biotechnology, sc-47778) https://www.scbt.com/p/beta-actin-antibody-c4?srsltid=AfmBOorYSdH6b3qXmpTQQ79i5ysFyCedgXl9lN6y-XAz_aQ-lk8eYvUs
Ki67 (Abcam, ab16667) https://www.abcam.com/en-us/products/primary-antibodies/ki67-antibody-sp6-ab16667
Cleaved caspase-3 (Abcam, ab2302) https://www.abcam.com/en-us/products/primary-antibodies/cleaved-caspase-3-antibody-ab2302
ZO-1 (Invitrogen, 61-7300) https://www.thermofisher.com/antibody/product/ZO-1-Antibody-Polyclonal/61-7300
ZO-1 (Invitrogen, 33-9100) https://www.thermofisher.com/antibody/product/ZO-1-Antibody-clone-ZO1-1A12-Monoclonal/33-9100
Bcl-2 (Abcam, ab32124) https://www.abcam.com/en-us/products/primary-antibodies/bcl-2-antibody-e17-ab32124
Bax (Santa Cruz Biotechnology, sc-20067) https://www.scbt.com/p/bax-antibody-2d2?srsltid=AfmBOoplhWVZCVftr8zGVTJr8TGpNVMd1ZR5_aPj8PwNboO0SjGc6BzT
Bcl-xL (Santa Cruz Biotechnology, sc-8392) https://www.scbt.com/p/bcl-xl-antibody-h-5?srsltid=AfmBOopedQOABJTPyT_fO84_f3RSCAWsoq5OlAI2mAmzr2w0Kxl_CNtX
p53 (Cell Signaling Technology, #2527) https://www.cellsignal.com/products/primary-antibodies/p53-7f5-rabbit-monoclonal-antibody/2527?srsltid=AfmBOoopM73exjzXaLA1Gtb8Pqg50xIU0Tz-POC1G1hk_uYYOVhK0odJ

Eukaryotic cell lines

Policy information about cell lines and Sex and Gender in Research

Cell line source(s)

ARPE-19 was purchased from American Type Culture Collection (ATCC, CRL-2302).

Authentication

The ARPE-19 cell line has been validated by the suppliers.

Mycoplasma contamination

ARPE-19 cells were mycoplasma free and determined by mycoplasma detection kit (LONZA, #LT-07-118).

Commonly misidentified lines (See ICLAC register) | N/A

Animals and other research organisms

Policy information about studies involving animals; ARRIVE guidelines recommended for reporting animal research, and Sex and Gender in Research

Laboratory animals	Male C57BL/6J mice were purchased from Jackson Laboratory (Orient Bio, Seongnam, Korea). All mice were maintained at the Konkuk University Laboratory Animal Research Center under Specific Pathogen-Free (SPF) conditions. All animal experiments were conducted according to the guidelines approved by the Institutional Animal Care and Use Committee (IACUC) at Konkuk University (approval No. KU22126).
Wild animals	N/A
Reporting on sex	All experiments were performed on male mice and are specified in methods and results.
Field-collected samples	N/A
Ethics oversight	All experimental and animal care procedures were performed according to guidelines approved by the Konkuk University (KU IACUC), Seoul, South Korea.

Note that full information on the approval of the study protocol must also be provided in the manuscript.

Plants

Seed stocks	N/A
Novel plant genotypes	N/A
Authentication	N/A

Flow Cytometry

Plots

Confirm that:

☒ The axis labels state the marker and fluorochrome used (e.g. CD4-FITC).

☒ The axis scales are clearly visible. Include numbers along axes only for bottom left plot of group (a 'group' is an analysis of identical markers).

☒ All plots are contour plots with outliers or pseudocolor plots.

☒ A numerical value for number of cells or percentage (with statistics) is provided.

Methodology

Sample preparation	Cells were cultured in standard growth medium and harvested by gentle detachment. After washing with PBS, cells were incubated with FITC-loaded samples, collected, and resuspended in PBS for flow cytometry analysis.
Instrument	Flow cytometry data were acquired using a BD FACSVerse flow cytometer (BD Biosciences).
Software	Flow cytometry data were analyzed using FlowJo software (BD Biosciences).
Cell population abundance	Cellular uptake was quantified as the mean fluorescence intensity (MFI) of the gated cell population.
Gating strategy	Cells were first gated based on forward and side scatter to exclude debris and select the main cell population. Background fluorescence was defined using unstained control cells, and positive populations were determined relative to this control.

☒ Tick this box to confirm that a figure exemplifying the gating strategy is provided in the Supplementary Information.
